# Children’s Dental Anxiety during the COVID-19 Pandemic: Polish Experience

**DOI:** 10.3390/jcm9092751

**Published:** 2020-08-25

**Authors:** Aneta Olszewska, Piotr Rzymski

**Affiliations:** 1 Department of Facial Malformation, Poznan University of Medical Sciences, 60-812 Poznań, Poland; 2 Department of Environmental Medicine, Poznan University of Medical Sciences, 60-806 Poznań, Poland; 3 Integrated Science Association (ISA), Universal Scientific Education and Research Network (USERN), 60-806 Poznań, Poland

**Keywords:** COVID-19, SARS-CoV-2, dental care, children, dentist-patient relation, pandemic

## Abstract

Dental fear and anxiety is a significant issue that affects pediatric patients and creates challenges in oral health management. Considering that the coronavirus disease 2019 (COVID-19) pandemic, along with its associated sanitary regime, social distancing measures and nationwide quarantines, could itself induce public fears, including in children, it is of great interest to explore whether this situation and the necessity of reorganizing dental care could potentially affect the emotional state of pediatric patients facing a need for urgent dental intervention. The present study assessed the emotional state of children ≤ seven years old (*n* = 25) requiring dental healthcare during a nationwide quarantine in Poland, as well as the anxiety levels of their caregivers. The Faces Anxiety Scale was adopted, and the evaluation was independently performed by the dentist, caregivers and children themselves. The level of anxiety in caregivers was also measured. As demonstrated, children requiring dental intervention during the nationwide quarantine did not reveal a significantly higher anxiety level as compared to the age- and indication-matched pre-pandemic control group (*n* = 20), regardless of whether their emotional state was evaluated by the dentist, caregivers, or by themselves. However, the share of children scoring the lowest anxiety level in all assessments was smaller in the pandemic group. Boys in the pandemic group had a higher anxiety level, as indicated by a caregiver assessment, and displayed a negative correlation with age in all three types of evaluation. Moreover, caregiver anxiety levels were higher in the pandemic group as compared to the pre-pandemic subset and revealed stronger correlations with the dental anxiety in children. The results suggest that the reorganization of oral healthcare under the pandemic scenario did not have a profound effect on children’s dental anxiety. Nevertheless, findings in young boys highlight that they may be more vulnerable and require special care to mitigate their anxiety and decrease the risk of dentophobia in the future—these observations must be, however, treated with caution due to the small sample size and require further confirmation. Moreover, it is important to reassure caregivers of the safety of the dental visit during the pandemic to minimize the effect of their own anxiety on dental fears in children.

## 1. Introduction

The outbreak of the novel coronavirus disease COVID-19 in December 2019 that spread across the Asian continent and eventually turned into a pandemic [1,2] created numerous challenges in health care sectors unrelated to the management of infectious diseases, including dentistry [3,4,5,6,7]. Following the confirmation of the first case in an increasing number of countries, the physical infrastructure, including entire hospitals, hospital wards, beds and technical equipment, had to be repurposed. The workforce resources in health care have also undergone reorganization and reallocation to support the response to the pandemic. This has led to the limitation or postponing of non-emergency health care appointments and treatments. In the meantime, the rapidly evolving epidemiological situation has forced numerous countries to implement strict sanitary regimes and social distancing measures, and eventually impose nationwide quarantines to decrease transmission rates. Under such circumstances, and particularly lockdown-associated isolation, significant public distress can be seen, further magnified by mass media coverage, often based on sensational, panic promoting headlines, as well as by online social media through which the spread of unsupported claims and fake news could be seen [8,9,10]. This stress is not only related to a fear of contracting the disease but also to significant and rapid changes to lifestyle and work [11]. As already demonstrated, all of these factors can be so profoundly affecting that a relevant percentage of individuals are put at risk of less or more severe mental health issues [12], including children and their parents [13,14,15,16]. Moreover, one study in adults has already reported that the introduction of the pandemic caused anxiety in 25% of dental patients [17]. However, no research has specifically addressed dental anxiety levels during COVID-19, including that in children.

During the COVID-19 pandemic, children suffer from not going to kindergartens and schools, not having real contact with their friends and some family members and having to live at home with decreased ability to practice physical activity and carry out some of their hobbies. As shown in an Italian survey, a significant percentage of children become nervous when hearing about the pandemic (e.g., on television) [18]. To protect them from distress, parents might often avoid discussing the pandemic, although the research supports that sensitive communication during the crisis has benefits for children’s wellbeing [19,20]. In addition, their emotional state can closely reflect that of caregivers, further adding to anxiety level [21].

Providing children with dental care during the COVID-19 pandemic, and in particular during the increased social restrictions, can be a challenging task. It is known that appointments are often met with dental fear and anxiety, while 7–8% of children in pre- and early school age display it at a level which might interfere with dental procedures [22,23]. This primarily originates from fears of procedure and pain [24,25,26,27], but under pandemic-related lockdown these emotions and feelings can potentially be further exacerbated by the new stressors present in their environment as well as by the psychological tension resonating from the caregivers who need to take a decision to leave home and potentially risk contracting SARS-CoV-2. Moreover, in the regular setting, there is a possibility of the effective management of children’s fear, anxiety and phobia with the support of caregivers and through desensitization, tell-show-do, positive reinforcement, and other behavioral techniques [26,28]. As the main route of SARS-CoV-2 transmission is via airborne droplets, dental staff are required to use personal protective equipment (PPE), i.e., suits, goggles, face visors, and face masks. The PPE affects the voice tone, makes it more difficult for children to understand what a dentist is communicating, does not allow children to read facial expressions which are important for building their trust with a dentist, adds to white coat syndrome, and overall hinders interaction with the patient [7]. Although some techniques to manage the anxiety level are still possible and still performed, the additional safety measures may effectively worsen the relationship between pediatric patients and personnel.

Survey studies reported that 50–70% of dental professionals admit to experiencing higher stress and anxiety levels as a result of the COVID-19 pandemic [29,30], an effect that may, in turn, alter their relationship with the pediatric patient. All of this, along with empty dental clinics and the smell of ozone disinfectant, can affect the children’s trust in and perception of the oral healthcare provider, particularly in patients with a high fear level, and potentially aggravate the possibility of reassuring and de-stressing them in the waiting room and dental office, and eventually this may reinforce dental anxiety.

The present study aimed to explore the level of anxiety in children and their caregivers during dental visits at the time of the nationwide quarantine in Poland. The first COVID-19 case in the country was confirmed on 4 March 2020. Following this, schools and universities were closed on 11 March and a nationwide quarantine was imposed on 24 March. The lockdown lasted till 4 May when hotels and shopping centers were permitted to reopen while on 6 May daycare centers and kindergartens were allowed to resume their activities. Despite the easing of social distancing rules, dental care will continue to be provided with new sanitary regimes. Evaluation of the emotional state of young patients and their caregivers during the pandemic and related nationwide lockdown is important to understand whether the additional safety measures can affect dental fear and to discuss the strategies that could effectively decrease this.

## 2. Experimental Section

### 2.1. Study Design

The study was designed to explore the emotional state of 25 children aged four–seven who required intervention at the Pediatric Dentistry Clinic at Poznan University of Medical Sciences, Poznań, Poland, during the nationwide quarantine in Poland (pandemic group), and to compare it to an age- and an indication-matched group of pediatric patients before the COVID-19 pandemic (pre-pandemic group). The pre-pandemic group used for comparison consisted of 20 children aged four–seven requiring dental intervention between January and June 2018. The level of anxiety related to the dental visit was also assessed in caregivers in both groups, as they remain in a close emotional relationship with their children and can affect each other. The following medical indications for dental appointments were considered in both groups of pediatric patients: tooth extraction, abscess, dental trauma (traumatic injury to orofacial tissues), mucosal lesion, and necessity of performing pulp treatment. All children enrolled in the study had a dental history of no more than three appointments, no history of chronic disease and no mental disorder. The following descriptive variables of the studied children were collected: age, sex, and medical indication for dental intervention.

The anxiety levels in the pandemic group was evaluated between 24 March and 30 April 2020 (pandemic group). At the time the study was initiated, COVID-19 infections were active in 195 countries and territories, including Poland, with 425,675 cases and 19,195 deaths confirmed globally. During the time the study was conducted, the total number of confirmed infections and fatal cases in Poland increased from 901 to 12,877 and from 10 to 644, respectively. This period represents the strictest national lockdown, which was imposed from 24 March with some restrictions lifted at the beginning of May when hotels, shopping centers, daycare centers, and kindergartens were permitted to reopen. During this period, the following modifications to dental healthcare procedures were undertaken:

1. To limit the direct time of the visit, medical history was taken via phone or online call.
2. Children’s caregivers were informed about new safety regulations and measures employed by the dental clinic, and about additional safety measures.
3. Before the appointment, all caregivers were advised to explain the safety measures to children and show them a picture of dental staff in PPE to make them more familiar with the situation.
4. The body temperature of each child and caregiver was measured at the entrance to the waiting room.
5. Each child and caregiver was instructed to disinfect hands in the waiting room.
6. Only one adult caregiver was allowed to accompany a child during the visit to the dental office.
7. In the case of all appointments, two professionals (a dentist and assistant) were maximally present in the dental office.

All caregivers and children in the pandemic group adhered to the above-mentioned rules.

The purpose and protocol of the study were explained to every child and their supervisor. Participation in the research was entirely voluntary. Prior to participation, all supervisors were asked to give their written informed consent. The supervisor accompanied the child during the emotional assessment. The study protocol was submitted to the Bioethical Committee at Poznan University of Medical Sciences - according to the performed evaluation, it lacked the characteristics of a medical experiment and, in line with Polish law and the Good Clinical Practice, it did not require specific approval by the Bioethics Committee.

### 2.2. Emotional State Evaluation

**Figure 1:**
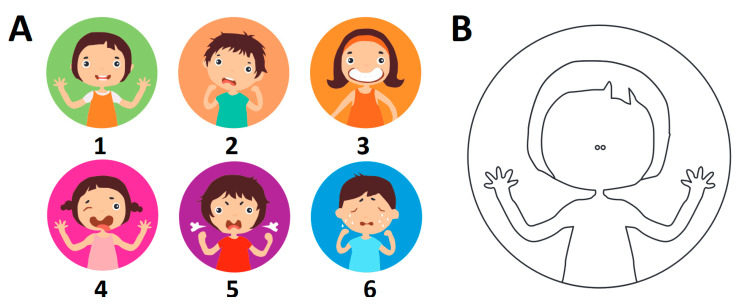
The graphical scale used to assess the emotional state of pediatric patients before the dental appointment (**A**) and a blank face (**B**) used by children to express their own emotional state by drawing missing elements: eyes, nose and lips.

The emotional state of children was evaluated in the waiting room prior to dental procedures by a caregiver, dentist and by the child. The whole procedure took max. 15 min. Dentists and caregivers independently assessed the children’s emotional state using the faces mood scale (Figure 1). The scale was prepared by a graphic artist according to the facial muscle changes involved in a fearful expression and based on photographs of faces showing increased fear [31]. The evaluating individual selected one of the six drawn faces that suited the child’s emotional state. The drawings were numbered as follows: 1—calm; 2—uncertain; 3—reserved, closed and uncooperative; 4—avoiding; 5—loud; 6—crying. The pediatric patients, given the paper with a blank face (Figure 1), were asked to complete the drawing by adding the facial elements: eyes, nose and lips. Prior to this, all children were instructed that a drawing should express their own emotions before the dental appointment. The dentist then categorized the children’s drawings to the corresponding faces 1–6, mostly by matching eye and lip expressions. Such a graphical approach in the evaluation of the emotional state of children appears to be advantageous compared to the numerical scale assessment - it only requires simplified verbal instructions and is more accessible for children to understand than a translation of their inner state to a particular numerical score [32]. It has also been demonstrated that children and their caregivers tend to prefer the faces scale over other evaluation methods [33,34]. The numbers associated with each drawing were operationalized by transforming them into 1–6 Likert scales, where 1 and 6 indicated the lowest and highest level of anxiety, respectively.

Additionally, the caregivers were asked to assess their own level of anxiety related to the dental visit by using a Likert scale 0–10, where 0 corresponded to lack of fear, 5 indicated medium anxiety, while 10 represented a very high level of anxiety.

### 2.3. Statistical Analysis

The statistical analyses were performed using Statistica v.13.1 (StatSoft Inc., Tulsa, OK, USA). Because age did not meet the assumption of Gaussian distribution (Shapiro–Wilk test; *p* < 0.05) and the emotional state of children was measured in the ordinal scale, non-parametric methods were employed. The differences in the emotional state scores in pandemic and pre-pandemic groups, as well as between boys and girls, were assessed with the Mann-Whitney U test. The association between children’s age and the scores were evaluated using Spearman’s rank correlation coefficient (Rs). The differences in the prevalence of medical indications for dental intervention in the pandemic and the pre-pandemic group were assessed by Pearson’s χ^2^ test. A value of *p* < 0.05 was considered statistically significant.

## 3. Results

The pandemic group consisted of 25 children: 15 boys (mean ± SD age 5.1 ± 1.1 years) and 10 girls (mean ± SD age 5.3 ± 0.9 years). The pre-pandemic group consisted of 10 boys (mean ± SD age 4.5 ± 0.8 years) and 10 girls (mean ± SD age 5.6 ± 1.1 years). The comparative age of the two groups did not differ (*p* > 0.05, Mann-Whitney U test). The demographic breakdown of medical indications for a dental visit in both groups is summarized in Table 1. The prevalence of these indications did not differ between the studied groups (*p* > 0.05, χ^2^ test in all cases); tooth extraction was the most frequent procedure (Table 2).

**Figure 2:**
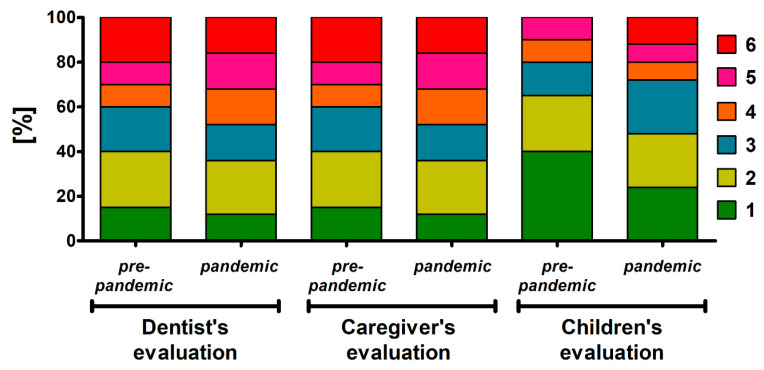
The emotional state of pediatric patients before the dental appointment in the pre-pandemic (*n* = 20) and pandemic group (*n* = 25) as assessed by dentists, caregivers and children themselves.

The emotional state of children in the pandemic group did not differ from that in the pre-pandemic group, either when assessed by the dentist [median (interquartile range, IQR): 3 (2–5) vs. 2 (1–4)], caregiver [3 (2–5) vs. 3 (2–5)], or the children themselves [3 (2–4) vs. 2 (1–3)] (*p* > 0.05 in all cases, Mann-Whitney U test). However, the percentage of children for whom the highest anxiety score of 6 in the pre-pandemic and pandemic groups was 20.0 and 12.0% (dentist’s evaluation), 20.0 and 16.0% (caregiver’s evaluation) and 10.0 and 24.0% (self-evaluation by children), respectively In turn, the lowest anxiety score of 1 in these groups was 30.0 and 16% (dentist’s evaluation), 15.0 and 12.0% (caregiver’s evaluation) and 40.0 and 24.0% (self-evaluation by children), respectively The summary of scores in each group given by dentists, caregivers and children is presented in Figure 2. The scores given by the dentist, caregivers, and self-reported by the children were all highly correlated in both studied groups of children (Table 2).

**Figure 3:**
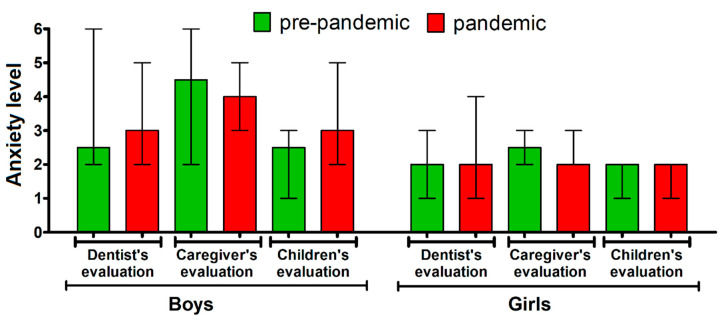
The emotional state of boys and girls before the dental appointment in the pre-pandemic (*n* = 20) and pandemic group (*n* = 25) as assessed by dentists, caregivers and children themselves. The bars represent median, the whiskers represent interquartile range.

Moreover, the gender of children did not differentiate the level of their anxiety in either pre- or pandemic groups (*p* > 0.05 in all cases, Mann-Whitney U test) (Figure 3). The only exception was the assessment of children’s anxiety performed by parents in the pandemic group, with a higher score given for boys than girls [median (IQR): 4 (3–5) vs. 2 (2–3)] (*p* < 0.05, Mann-Whitney U test). The comparison of each gender across the two groups yielded no differences in anxiety level (*p* > 0.05 in both cases, Mann-Whitney U test).

However, as presented in Table 3, the percentage of boys in the pandemic group having the lowest and highest anxiety levels was respectively decreased and increased compared to the pre-pandemic subset in all three evaluations—such a phenomenon was not seen in the case of girls.

As shown in Table 4, a number of negative correlations were found between children’s age in the pandemic group and the scores they were given, including their own assessment of emotional state. However, when differentiated by gender, these correlations were only significant for the subset of boys (Table 4). When evaluated by the dentist, caregivers and children themselves, the median (IQR) scores of anxiety in boys aged four from the pandemic group were 5 (5–6), 5 (5–6) and 4 (3–6), respectively, while for those aged seven, these scores were 1 (1–2), 2 (1–4) ad 2 (1–4), respectively.

A significant difference in the anxiety of caregivers accompanying children before and during the pandemic was observed with a median (IQR) level of 6 (4–8) and 3 (2–4), respectively (*p* < 0.01, Mann-Whitney U test). The number of caregivers indicating a score >5 (above the level of anxiety defined as ‘medium’) in pre-pandemic and pandemic groups was 1/20 (5%) and 13/25 (52%), respectively. The parental anxiety level in the pandemic group was positively correlated with children’s dental anxiety as assessed by the dentist (Rs = 0.80, *p* < 0.05), the caregiver (Rs = 0.76, *p* < 0.05) and self-evaluated by the children (0.74, *p* < 0.05). In the pre-pandemic group, positive correlations with the children’s anxiety evaluated by caregivers (Rs = 0.57, *p* < 0.05) and children themselves (Rs = 0.72 *p* < 0.05) were observed.

## 4. Discussion

The present study is the first to report on the emotional state of children ≤ seven years during the COVID-19 pandemic. Its unique aspect is that it was specifically conducted during the strictest form of the nationwide quarantine. The previous research in adults has shown that dental patients reported feeling anxious about the pandemic, although this finding cannot be attributed directly to dental anxiety, and, contrary to our study, it did not investigate anxiety levels during the dental appointment [17]. In turn, dental fear and anxiety in children represent an important issue in dental management, and as hypothesized, fears related to the ongoing epidemiological situation and associated changes in the dental service organization could further potentiate them. This could be particularly expected given the fact that the emotional state of children is often influenced by that of their parents [35,36], and during the pandemic-related lockdown the fear of contracting SARS-CoV-2 during different activities, e.g., while shopping in the grocery store, were frequently reported [11]. On the other hand, the additional measures undertaken to shorten dental appointments via applying telemedicine, i.e., video/phone consultation to take medical history and talk through the proposed treatment, as well as efforts to make children as familiar as possible with the new sanitary regime during the dental visit, could possibly mitigate, at least to some extent, fear and anxiety in children. The observations of the present study indicate that children requiring dental intervention during the COVID-19 pandemic-related nationwide quarantine may not experience significant changes in their emotional state, as compared to the pre-pandemic, age- and indication-matched group. It should be however noted that the share of patients with the lowest anxiety level in all assessments was smaller in the pandemic group.

The emotional state of pediatric patients was evaluated in the present study by children themselves, but also by their caregiver and the dentist. Such an approach allows for a broader assessment of the child’s inner state. As previously suggested by studies on pain intensity, self-reporting tools can be inaccurate for children younger than seven years due to poor understanding of the method and the skills needed to express their experiences not yet being fully developed [37,38]. Therefore, in such groups, complementary observational measures should be employed. In such a case, caregivers can be used as a proxy for patients’ reports, especially in situations in which some communication barriers may exist. Finally, the assessment of the child by the dentist is also valuable as it is based on professional experience and not biased by the strong emotional relationship that exists between a child and a parent [39]. One should, however, note that a survey conducted in 30 countries reported increased levels of anxiety in dentists during the COVID-19 pandemic [29], and this may potentially alter their perception and assessment of the patient’s emotional state. Importantly though, the present study demonstrated that the results on children’s emotional state obtained using the face scale from the dentist, caregivers and self-reported by the pediatric patients generally agree with each other.

Previous research has related dental anxiety in children to various factors such as personality traits, increased general fears, a history of painful dental experiences, parental dental fears and other family-related factors [22,40,41]. Some studies have also found that it tends to be higher in girls than boys, and in younger children. These age and gender-related differences were, however, not always confirmed [42]. In the present study, gender-differences, with a higher level of anxiety in boys, were observed only in the group undergoing a dental procedure during the pandemic-related nationwide quarantine, and only when the assessment was performed by a caregiver. One should note that the percentage of boys in the pandemic group who displayed the highest and lowest level of anxiety increased and decreased, respectively, when compared to the pre-pandemic subset. Furthermore, the present study suggests that younger boys were the most vulnerable, as highlighted by the negative correlation of all anxiety assessments and their age, and the highest scores seen in patients aged 4. This is an interesting finding since general anxiety levels tend to be higher in girls during early childhood [43]. It can be hypothesized that under the pandemic scenario it may be more challenging to explain the nature of the situation to younger boys than girls, due to the difference in the development of their language skills [44,45,46,47]. As reported, four-year-old boys reveal a significantly lower expression of pivotal factors for human communication, such as FOXP2 protein [48]. Nevertheless, the present results highlight that special care may be needed for younger boys requiring a dental intervention during pandemic in order to mitigate their anxiety and decrease the induction of potential dentophobia in the future. One should however stress that implementation of any mitigation strategies in this regard should only be considered if the present results, based on small sample size, would find confirmation in future studies.

Importantly, the present study clearly shows that parental anxiety levels are correlated with the emotional state of children and that this association was stronger during the pandemic. The percentage of caregivers having anxiety above the medium level was over 10-fold higher in the pandemic group when compared to the pre-pandemic subset. Increased anxiety in adults during epidemiological events is not uncommon and has been observed previously, e.g., during the H1N1 pandemic in 2009–2010 [49,50,51]. Unsurprisingly, it has also been reported during the COVID-19 pandemic [52,53,54]. 

In turn, previous dental studies show that parents play a key role in children’s anxiety and development [55,56,57]. Therefore, it is of high importance, particularly during the pandemic, to ensure that caregivers of pediatric patients are fully aware of these links and to educate them on how to make a dental visit a more comfortable event for children. Considering that parental anxiety levels in the pandemic group were higher than in the pre-pandemic group, it can be suggested that fears related to COVID-19 and contracting SARS-CoV-2 were responsible for this effect. It is, therefore, necessary for healthcare professionals to explain, prior to the appointment, all the measures undertaken to maximally limit the risk of infection in the dentist’s surgery and to reassure patients of their safety. This can be achieved with a phone or video call preceding the dental visit during which medical history is also recorded.

Although the present study reports on the important issue of dental healthcare under a pandemic scenario, the study limitations must be emphasized. Firstly, the results represented a single-center experience and were obtained from a small population size. Moreover, dental anxiety in children is multifactorial, and this research did not explore the effect of personal traits and family-related issues, including socioeconomic status and level of education of caregivers [40,41,58,59,60]. Moreover, transforming the Faces Anxiety Scale into the Likert scale allowed for a more in-depth statistical elaboration of the results, although the study was not designed to delve into explaining the exact reasons for the observed anxiety levels in children. This would require additional questions, time spent in the dental clinic, and communication approaches that were challenging to apply during the dental clinical practice under the scenario of the most strict form of the COVID-19 related quarantine.

## 5. Conclusions

This is the first study to report on dental anxiety in children during the strictest form of the COVID-19 lockdown. In general, the present research indicates that, contrary to the concerns that pediatric children will be significantly more stressed due to dental appointments during a nationwide quarantine related to the COVID-19 pandemic, their anxiety levels, assessed by the dentist, caregivers and by themselves, did not differ from the pre-pandemic group of pediatric patients. Nevertheless, the percentage of children having the lowest level of anxiety in all employed assessments decreased in the pandemic group. The results suggest that younger boys may potentially be more vulnerable in this regard—this finding should be treated with caution and would require further confirmation in larger-scale studies. Moreover, parental anxiety levels were highly associated with the emotional state of children, particularly during the pandemic period. It seems reasonable to make parents aware of this association and, further, to reassure their safety by explaining that the risks of contracting an infectious agent during a dental visit are mitigated by appropriate measures.

## Figures and Tables

**Table 1:** Spearman’s rank correlation coefficient calculated for the level of anxiety in the pre-pandemic (*n* = 20) and pandemic group of children (*n* = 25) before the dental appointment assessed by the dentist, caregivers and children themselves. All values are statistically significant.

	Group	Caregiver Evaluation	Children Evaluation
**Dentist evaluation**	pre-pandemic	0.88	0.59
	pandemic	0.86	0.75
**Caregivers evaluation**	pre-pandemic	-	0.79
	pandemic	-	0.82

**Table 2:** Medical indications for the dental visit in pediatric patients in the pandemic and pre-pandemic groups included in the present study.

Medical indication	Pandemic Group (*n* = 25)	Pre-pandemic Group (*n* = 20)
Tooth extraction (n/%)	14/56	10/50
Abscess treatment (n/%)	5/20	1/5
Mucosal lesion (n/%)	1/4	2/10
Pulp treatment (n/%)	2/8	5/25
Dental trauma (n/%)	3/12	2/10

**Table 3:** The percentage of boys and girls with the lowest and highest anxiety levels before the dental appointment in the pre-pandemic (*n* = 20) and pandemic group (*n* = 25) as assessed by dentists, caregivers and children themselves.

Evaluation	Score	Boys		Girls	
		Pre-Pandemic (*n* = 10)	Pandemic (*n* = 15)	Pre-Pandemic (*n* = 10)	Pandemic (*n* = 10)
Dentist (%)	Lowest	20.0	6.7	40.0	30.0
	Highest	10.0	13.3	10.0	10.0
Caregiver (%)	Lowest	10.0	6.7	20.0	20.0
	Highest	10.0	20.0	10.0	10.0
Children (%)	Lowest	40.0	13.3	40.0	40.0
	Highest	10.0	13.3	10.0	10.0

**Table 4:** Relationship between the age of pediatric patients and their fear as evaluated by dentists, caregivers and self-reported by children (Spearman’s rank correlation coefficient).

	Group	Dentist Evaluation	Caregiver Evaluation	Children Evaluation
**Age**	Pandemic boys (*n* = 15) girls (*n* = 10)	−0.67 * −0.84 * −0.34 ^ns^	−0.63 * −0.69 * 0.57 ^ns^	−0.44 * −0.57 ^*^ −0.39 ^ns^
	Pre-pandemic boys (*n* = 10) girls (*n* = 10)	−0.25 ^ns^ −0.08 ^ns^ −0.38 ^ns^	−0.40 ^ns^ −0.14 ^ns^ −0.69 *	−0.36 ^ns^ −0.32 ^ns^ −0.52 ^ns^

* *p* < 0.05; ns—not significant (*p* > 0.05).
